# Medical Items

**Published:** 1912-12

**Authors:** 


### MEDICAL ITEMS

Every physician who has any considerable obstetrical practice owes it to himself and to his patients to familiarize himeslf with oxytocic function of Pituitrin. Here is an agent which, according to reports in the medical journals of the Old World (notably of Germany)—if obstetricians adopt it generally, as now seems likely—is destined to rob childbirth of much of its pain and terror. What shall we say of such an agent that fails but once in over a hundred cases in which it is used? And that is just what happened in Dresden, according to a report of Vogt, of Royal Gynecological Clinic of that city. Vogt adds: "It was not necessary to have recourse to forceps in a single instance in which Pituitrian was employed."

For the benefit of physicians who are uninformed on the subject, it may be said that Pituitrin is an extract of the posterior or infundibular portion of the pituitary gland. While n use for a number of years—chiefly, perhaps, as a hemostatic and heart stimulant—it is only of late, comparatively speaking, that its value in uterine inertia has been fully understood. The product is prepared and marketed by Parke, Davis & Co., to whom inquiries should be addressed for further particulars of this remarkable agent. Not very long ago the company issued a pamphlet in which a number of interesting and surprising case reports were published. We understand that copies of this Pituitrin pamphlet are still availablee and may be obtained upon application to Parke, Davis & Co., at ttheir general offices in Detroit, Michigan.

